# Molecular and Biological Characterization of a New Isolate of Guinea Pig Cytomegalovirus

**DOI:** 10.3390/v6020448

**Published:** 2014-01-27

**Authors:** Mark R. Schleiss, Shane McAllister, Anibal G. Armién, Nelmary Hernandez-Alvarado, Claudia Fernández-Alarcón, Jason C. Zabeli, Thiruvarangan Ramaraj, John A. Crow, Michael A. McVoy

**Affiliations:** 1 Division of Pediatric Infectious Diseases, Department of Pediatrics, University of Minnesota Medical School, Minneapolis, MN 55455, USA; E-Mails: smcallis@umn.edu (S.M.); hernande@umn.edu (N.H.-A.); ferna128@umn.edu (C.F.-A.); zabe043@umn.edu (J.C.Z.); 2 Department of Veterinary Population Medicine and Veterinary Diagnostic Laboratory, College of Veterinary Medicine, Saint Paul, MN 55108, USA; E-Mail: armie001@umn.edu; 3 National Center for Genome Resources (NCGR), Santa Fe, NM 87505, USA; E-Mails: tr@ncgr.org (T.R.); jac@ncgr.org (J.A.C.); 4 Division of Pediatric Infectious Diseases, Department of Pediatrics, Virginia Commonwealth University School of Medicine, Richmond, VA 23298, USA; E-Mail: mmcvoy@vcu.edu

**Keywords:** guinea pig cytomegalovirus, cytomegalovirus strain variation, CMV immune evasion, congenital cytomegalovirus infection, congenital CMV vaccines

## Abstract

Development of a vaccine against congenital infection with human cytomegalovirus is complicated by the issue of re-infection, with subsequent vertical transmission, in women with pre-conception immunity to the virus. The study of experimental therapeutic prevention of re-infection would ideally be undertaken in a small animal model, such as the guinea pig cytomegalovirus (GPCMV) model, prior to human clinical trials. However, the ability to model re-infection in the GPCMV model has been limited by availability of only one strain of virus, the 22122 strain, isolated in 1957. In this report, we describe the isolation of a new GPCMV strain, the CIDMTR strain. This strain demonstrated morphological characteristics of a typical *Herpesvirinae* by electron microscopy. Illumina and PacBio sequencing demonstrated a genome of 232,778 nt. Novel open reading frames ORFs not found in reference strain 22122 included an additional MHC Class I homolog near the right genome terminus. The CIDMTR strain was capable of dissemination in immune compromised guinea pigs, and was found to be capable of congenital transmission in GPCMV-immune dams previously infected with salivary gland‑adapted strain 22122 virus. The availability of a new GPCMV strain should facilitate study of re-infection in this small animal model.

## 1. Introduction

Development of a vaccine against human cytomegalovirus (HCMV) is a major public health priority [1]. The suggestion that passively transferred antibody protects the fetus against infection and injury [2] has driven efforts to develop recombinant subunit vaccines targeting major envelope glycoproteins, such as glycoprotein B (gB) [3,4]. Although clinical trials of recombinant gB vaccines have shown some degree of effectiveness in preventing HCMV infection and disease in high risk populations [5,6], vaccine mediated protection with vaccines targeting this single envelope glycoprotein appears to be incomplete. Moreover, the effectiveness of natural immunity in preventing congenital HCMV infection and its attendant sequelae is itself a matter of some controversy. A number of recent studies have described fetal HCMV transmission in women with preconception immunity, due to re-infection with new strains of HCMV [7,8,9,10,11,12]. Such infections can produce sequelae identical to those observed in congenitally infected infants born to women with primary HCMV infection in pregnancy [13,14]. These observations certainly complicate HCMV vaccine design, and suggest that: (1) for full protection, an HCMV vaccine may need to elicit responses superior to those conferred by natural immunity; (2) there may be a strong rationale for vaccinating women of childbearing age who are already HCMV seropositive, in addition to targeting and immunizing seronegative women, toward the goal of preventing re-infection with subsequent transmission of the “new” strain. 

Several clinical studies have documented the phenomena of re-infection in women of childbearing age. In one prospective study performed at the University of Alabama-Birmingham, serum specimens from 46 women with preconceptional immunity against HCMV obtained during a previous pregnancy and a new pregnancy were analyzed for antibodies against the strain-specific epitopes of HCMV glycoprotein H (gH), and the nucleotide sequences of the gH gene from seven HCMV isolates were determined. Ten of the 16 mothers with infected children (62%) acquired new antibody specificities against gH, as compared with only 4 of the 30 mothers of uninfected infants (13%), suggesting that acquisition of an infection with a virus expressing a novel strain-specific gH genotype during pregnancy was associated with congenital transmission [9]. In another study in Brazil that followed 7,848 women prospectively, sera from 40 mothers of congenitally infected infants and 109 mothers of uninfected control newborns were analyzed for strain-specific anti-HCMV antibodies, based not only on polymorphisms within gH binding sites, but also a second antibody reactivity site on gB [14,15]. Seven of 40 (17.5%) study women, but only 5 of 109 (4.6%) controls acquired antibodies reactive with new HCMV strains during pregnancy (*p* = 0.002), suggesting that maternal reinfection by new strains of HCMV is a major source of congenital infection in this population. In a study of re-infection (based on acquisition of new gB and/or gH antibody specificities) in 205 seropositive women performed by Ross and colleagues at UAB, approximately one-third of the study participants (59 of 205) were noted to have reinfection, using this definition, during follow-up [10]. The molecular and immunological correlates of re-infection are unclear. There is some evidence that gB polymorphisms in clinical isolates may be less important for re-infection than polymorphisms in gH and other envelope glycoproteins. In a study in Brazil, infections in immunocompetent women with strains corresponding to more than one gB genotype were not common [16]. Additionally, in a study of the HCMV strains acquired longitudinally in women who developed infection in spite of being enrolled in the recombinant gB vaccine trial at UAB [5], there was no selection for or against any non-vaccine gB subtype, in spite of women being immunized only with gB protein corresponding to the Towne (gB1 subtype) strain [17]. Other evidence suggests that the response (or lack thereof) to the envelope glycoprotein N (gN) may play a role in predisposing to re-infection with new HCMV strains expressing heterologous gB and/or gH genotypes [18,19,20]. 

Irrespective of the mechanism(s) involved, the issue of re-infection is a major challenge in vaccine design. There is increasing evidence that congenital HCMV infections after nonprimary maternal infections can lead to symptomatic disease and substantial long-term sequelae. Notably, recent evidence from a study at UAB indicated that the incidence of hearing loss in infants infected after nonprimary maternal infection was similar to that in infected infants born to women with primary infection [13], although in this study, infants in the primary infection group were more likely to demonstrate progressive and severe or profound hearing loss, compared to infants in the non-primary group. 

Since the consequences of re-infection and congenital transmission with a newly acquired strain in pregnant seropositive women can be similar to those that occur after primary infection and transmission in CMV-seronegative women [10,11,13,14], the study of re-infection in a small animal model of congenital transmission would be very useful for the modeling of vaccine strategies to prevent maternal re-infection [21,22]. Ideally, HCMV reinfection would be studied in an animal model prior to clinical vaccine trials. Unfortunately, the strict species-specificity of cytomegaloviruses precludes preclinical testing of HCMV vaccines in animals. However, a number of rodent and primate cytomegaloviruses are useful in modeling HCMV vaccines and therapies, given the conservation of many immunogenic structural proteins amongst the various viruses [23,24,25]. 

Among the small animal models, the guinea pig cytomegalovirus (GPCMV) is uniquely useful, since, in contrast to other rodent models, transplacental infection of the fetus occurs following viral challenge during pregnancy [24,26]. Hence, the GPCMV model is particularly well-suited to the study of vaccines against congenital infection. However, until now this model has relied exclusively on a single strain of GPCMV, 22122, isolated by Hartley in 1957 [27]. While it has been feasible to study re-infection by experimentally inoculating strain 22122 virus into naturally seropositive guinea pigs [28], the lack of defined genetic information on other GPCMV strains (e.g., those presumably latent in the seropositive animals used in the above mentioned studies) has made it impossible to study of the role of strain variation in fetal infection in the setting of preconception immunity. 

In this report, we describe the isolation of a novel strain of GPCMV, the CIDMTR strain. This communication represents the first report of detailed characterization of a GPCMV isolate since the original publication of isolation of the 22122 strain [27]. Although sequence and ORF structure were generally well conserved with the 22122 strain of GPCMV, the CIDMTR strain demonstrated some differences in genome structure, particularly in the right-hand end of the viral genome. There are also substantive differences in some protein coding sequences between the two strains, including sequences in envelope glycoproteins, suggesting that these proteins may have been the targets of immune selection during the evolution of GPCMV in the guinea pig host. We describe in this report the morphology and DNA sequence of this newly isolated strain, and report preliminary experiments regarding its pathogenesis *in vivo*. The availability of a second strain of GPCMV should enable the study of re-infection and, potentially, the development of vaccine strategies designed to protect against maternal re-infection in the guinea pig model of congenital cytomegalovirus infection.

## 2. Results and Discussion

### 2.1. Isolation of the CIDMTR Strain

**Figure 1:**
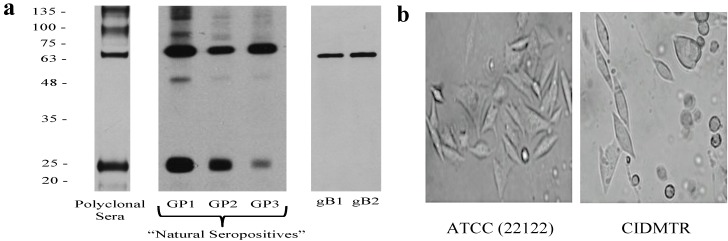
Western blot analysis of sera from three “naturally seropositive” commercially purchased outbred Hartley guinea pigs. (**a**) Western analysis using a pooled, high-titer polyclonal anti-GPCMV antisera from guinea pigs immunized with adjuvanted viral particles [30] or sera from three “natural seropositive” guinea pigs (GP1, GP2, GP3) obtained from a commercial supplier, using sucrose gradient-purified virions from 22122 strain as target antigen. Two independently derived, monospecific anti-GPCMV gB antibodies (moab 29-29 [31] (gB1) and moab IE321 (gB2)) are included as controls. (**b**) Photomicrographs comparing CPE of low-passage (P_2_) CIDMTR virus (right panel) to GPCMV strain 22122 (left panel) following infection of GPL cells. CIDMTR plaques are more rounded, with elliptical appearance, compared to the more spindle-like morphology of 22122-infected fibroblasts. Plaques photographed at 60× magnification.

In the course of ongoing vaccine and pathogenesis studies, guinea pigs were screened at the time of purchase for GPCMV antibodies, using an ELISA based on GPCMV strain 22122 [29]. Within a group of 24 guinea pigs purchased from a commercial source, 5 (21%) were found to be GPCMV-seropositive by ELISA. Western blot analysis was performed using sera from commercially purchased, ELISA-positive animals using purified strain 22122 virions as the source of target antigens (Figure 1). These studies confirmed that sera from these animals were broadly cross-reactive with GPCMV strain 22122 virion-associated polypeptides, suggesting that these animals were infected with GPCMV strains that were closely related antigenically to strain 22122. 

One of the seropositive animals identified in these preliminary experiments was immunosuppressed with cyclophosphamide, 100 mg/kg [32], and seven days later the animal was sacrificed and salivary gland (SG) homogenates harvested. Presumptive virus in the SG homogenate was passaged *in vivo* by inoculation of two GPCMV seronegative inbred strain 2 guinea pigs with 1 mL SG homogenate by subcutaneous route in the dorsal neck. Both of these animals demonstrated DNAemia (3.2 and 6.9 × 10^3^ genomes/mL of blood, respectively) at 14 days post-inoculation. Three weeks following inoculation, these animals were also immunosuppressed with 100 mg/kg of cyclophosphamide. One week later, these animals were sacrificed and SG homogenates were cultured on guinea pig lung fibroblast cells (GPLs). Eleven days after inoculation plaques with characteristic cytopathic effect (CPE) were observed in one of the SG homogenate cultures (designated P_0_). Supernatant from this flask was used to inoculate GPLs to generate a P_1_ stock of virus. When CPE was extensive (approximately 2 weeks later), an aliquot was removed for electron microscopy (EM) studies (described below, Section 2.2) and the remainder of this flask was expanded for large-scale virus propagation (20 flasks). These flasks were then incubated for an additional week prior to harvest of P_2_ viral stock (Figure 1b). Some of these infected flasks were used for DNA purification for Illumina MiSeq and Pacific Biosciences PacBio RS sequencing, as described below. The remainder of this stock was used for *in vivo* challenge experiments in guinea pigs, as described in Section 2.4.

### 2.2. Morphological Analyses by EM

**Figure 2:**
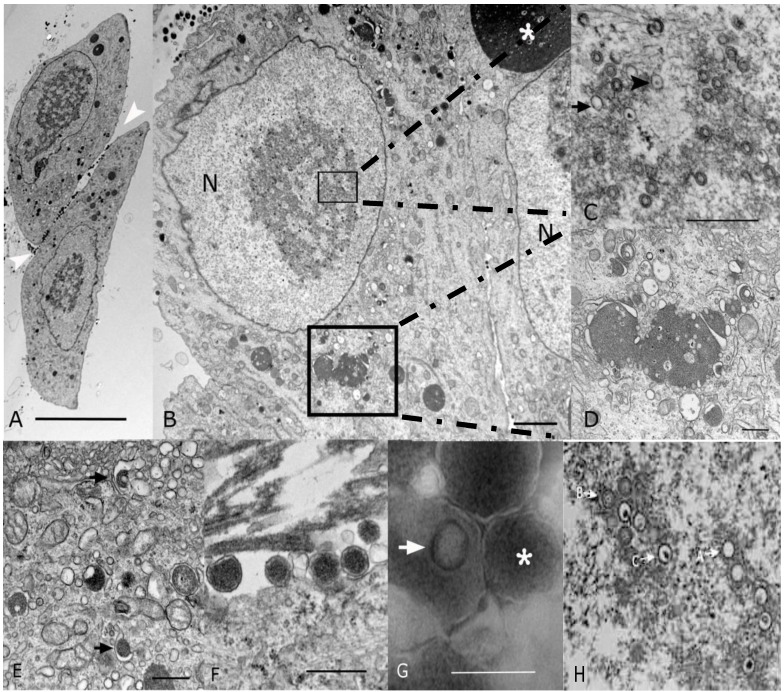
Transmission electron microscopy (EM) microphotographs of fibroblasts infected with the CIDMTR Strain of GPCMV. (**A**) Plastic embedded preparation contrasted with uranyl acetate/lead citrate of two enlarged fibroblasts showing cell intranuclear and cytoplasmic viral inclusions and numerous dense bodies (white arrowheads) in the intercellular space (bar = 10 µm). (**B**) Magnification of another CIDMTR-infected cell revealed nuclear (N) viral replication and nucleocapsid assembly sites (small square), as well as a large maturation site (asterisk) and dense body formation in the cytoplasm (large square, bar = 2 µm). (**C**) Additional magnification in which replication and capsid assembly in the nucleus (small square) is appreciated; note empty capsids (arrow) and DNA containing nucleocapsids (arrowhead; bar = 0.5 µm). (**D**) Magnification of virus maturation sites within the cytoplasm (large square in panel (**B**)) reveals electron dense material formed by aggregation of nucleocapsids and cell organelles such as Golgi systems, endoplasmic reticulum and vesicles (bar = 0.5 µm). (**E**) Capsid is demonstrated becoming coated with tegument proteins and then acquiring its final envelope by budding into vesicles (arrows; bar = 0.5 µm). (**F**) Several dense bodies are present in the intercellular space (bar = 0.5 µm). (**G**) Negative contrast preparation of enveloped B-capsid (arrow) and dense body (asterisk, bar = 0.5 µm) is demonstrated. (**H**) EM of strain 22122-infected cells demonstrating virtually identical morphology; A, B and C capsids are identified as described in text.

To confirm that the isolated virus had morphological characteristics of a cytomegalovirus, EM was performed. Analysis of P_1_ virus stock by conventional microscopy (Section 2.1) revealed 20% of the cells showed cytopathic effect. Cells were enlarged with large intranuclear and intracytoplasmic basophilic inclusions visible upon examination by light microscopy on thick section of plastic preparation stained with Toluidine blue (data not shown). Ultrastructurally, both intranuclear and intracytoplasmic inclusions were noted, interpreted as representative of nuclear and cytoplasmic viral factories (Figure 2A,B). Nuclear inclusions were typical for the center of replication and assembly characteristic of *Herpesvirinae* (Figure 2C). These nuclear factories showed a large amount of fibrillary electron dense material admixed with moderate to large numbers of empty A-capsids, scaffold-containing B-capsids, and DNA-containing C-capsids that are characteristic of herpesvirus capsid formation and genome packaging (Figure 2C; most capsids in this image are B-capsids). By comparison, a virtually identical appearance was noted in EMs from strain 22122-infected fibroblasts (Figure 2H). First envelopment was acquired at the inner nuclear membrane. The EM morphology supports a model whereby de-envelopment of nucleocapsids takes place at the outer nuclear membrane during egress, with the second envelopment and tegumentation occurring in the cytoplasm factory (Figure 2D). Rearrangement of Golgi cisterns, Golgi vesicles, multivesicular body and endoplasmic reticulum in conjunction with formation of nucleocapsids produced large aggregations that were often mixed with electron dense material. Numerous dense bodies were present in the intercellular space (Figure 2A,F). On negative contrast preparation, a large number of non-infectious enveloped particles (220.24 ± 48.15 nm) and dense bodies (357.83 ± 97.85 nm) were observed (Figure 2G). Infectious mature virions consisted of an envelope containing a 116.5 ± 2.68 nm in diameter icosahedral capsid with capsomeres of approximately 11.66 ± 1.87 nm. The CIDMTR strain of GPCMV presented similar cytopathic and pathogenic effects as those demonstrated by ATCC strain 22122 [33,34,35]. Nevertheless, some differences were noted between these two strains. The ATCC strain in contrast to the CIDMTR strain, demonstrated more efficient infection of fibroblasts, even at the same multiplicity of infection (MOI; MOI of 1), with over 80% of fibroblastic cells exhibiting cytopathic effect. The nuclear and cytoplasmic factories were less pronounced in the CIDMTR-infected cells, with generation of reduced quantities of infectious and non-infectious enveloped viral particles and dense bodies (data not shown).

### 2.3. Sequencing and Sequence Analysis

**Figure 3:**
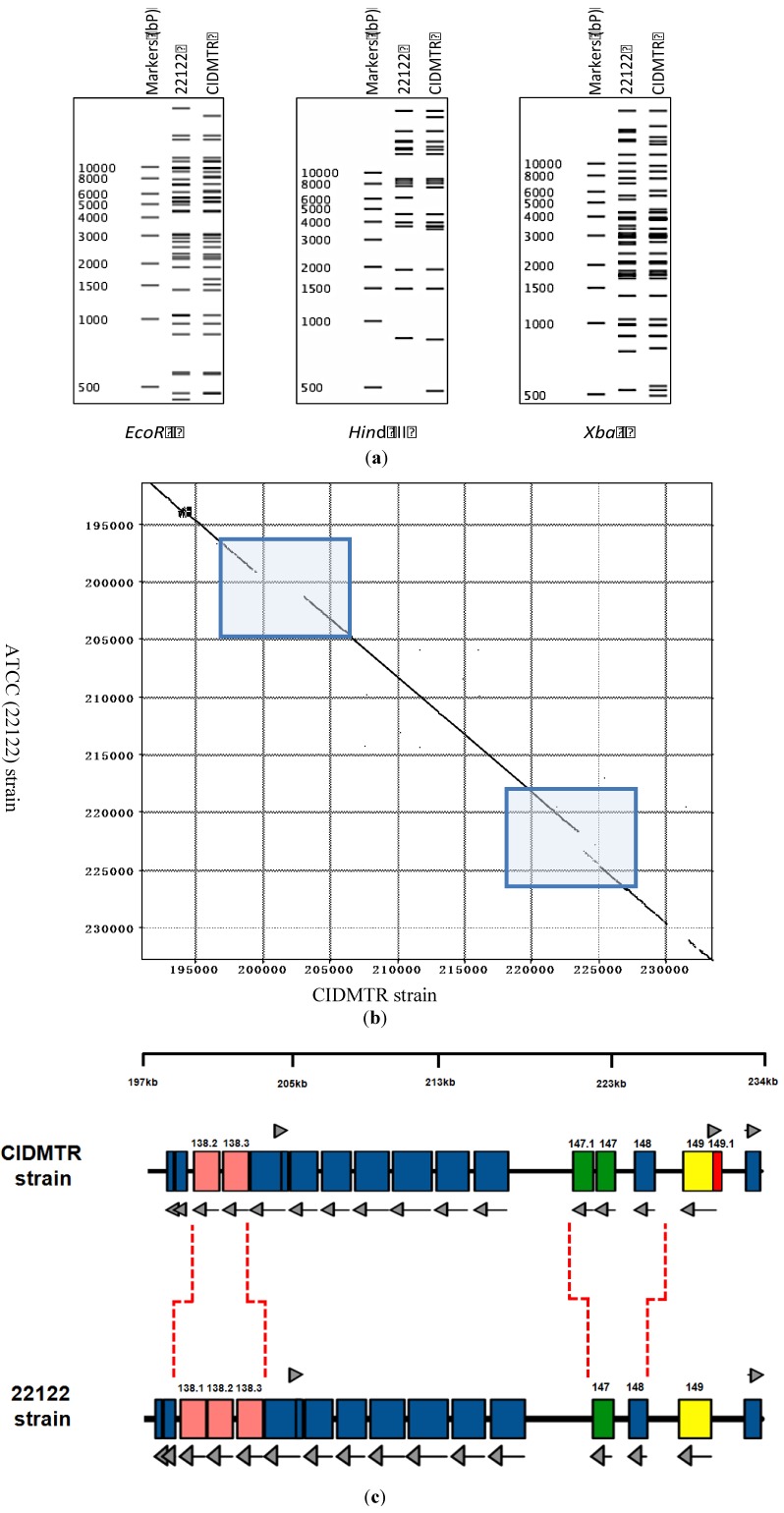
Strain-specific genome structure differences between CIDMTR and 22122. (**a**) *In silico* analysis of predicted restriction endonuclease profiles for CIDMTR strain and 22122 (ATCC) strain with enzymes *EcoR*I, *Hin*dIII, and *Xba*I. Predictions and profile generated using program CLC Main Workbench 6 [40]. (**b**) Dot-matrix comparison of CIDMTR *vs.* 22122 genomic sequences. Two genome regions demonstrating sequence variability resulting in ORFs unique to each strain are indicated by shaded boxes. (**c**) Linear map comparison of the major areas of genome discontinuity. Linear maps prepared using XPlasMap [41]. Colored boxes in blue represent predicted conserved ORFs. Light red and green colored ORFs demonstrate discordance between the two strains. In the CIDMTR strain, an additional ORF not annotated in 22122, 149.1, is also noted (dark red). Dashed lines indicate the positions where the sequences differ. See Table 1 for a full list of sequenced gene annotations.

Viral DNA (P_2_) was purified from the CIDMTR strain after two passages in GLFs and subjected to sequence analysis using the MiSeq and PacBio platforms as described in Section 3. The CIDMTR genome was 232,778 nt in length. Overall sequence homology to strain 22122 (accession #KC503762.1) was 98%, consistent with the hypothesis suggested by immunoblotting that CIDMTR is a GPCMV and not a novel and distinct betaherpesvirus of guinea pigs. As discussed further in Section 4, a possibility existed that genetic diversity of GPCMV isolates could be restricted due to bottlenecks in the breeding history of Hartley guinea pigs, from which both 22122 and CIDMTR are derived. Historically, HCMV strains were differentiated on the basis of restriction pattern polymorphisms. Epidemiologically unrelated isolates, considered unique HCMV strains, exhibit predominantly identical restriction patterns with occasional unique fragments. To compare CIDMTR to 22122 in these terms, genomic sequences for each virus were used to generate *in silico*-predicted restriction patterns for three enzymes (*Eco*R I, *Hin*d III, and *Xba* I). As shown in Figure 3a, the majority of fragments matched between the two virus genomes in all three predicted restriction enzyme patterns, while for each restriction pattern a few unique (unmatched) fragments were evident. These results are consistent with CIDMTR and 22122 being distinct GPCMV strains in a manner similar to that of HCMV strains. 

Sequence comparison revealed three regions of striking discrepancies between the two genome sequences (Figure 3b). The first was located between nucleotides 199227 and 203071 of the 22122 sequence. Within this 3,845-bp region, 1,437 bps were entirely missing from the CIDMTR genome, while the remainder corresponded with a poorly conserved (50% average nucleotide conservation) 2,366-bp region in CIDMTR. As a result, one of the three *gp138* family ORFs previously annotated in 22122 was missing from CIDMTR (Figure 3c). Curiously, this region corresponds to a hot-spot for spontaneous deletions that occur in response to over length genomes in GPCMV that also roughly corresponds to regions of instability/rearrangements in HCMV and rhesus cytomegalovirus [36].

The second region of discrepancy was located between nucleotides 221608 and 224743 of the CIDMTR sequence. Within this 3,136-bp region, 1,424 bps were entirely missing from the 22122 genome, and the remainder correspond with a poorly conserved (68% average nucleotide conservation) 1,684-bp region in 22122. Surprisingly, the additional sequences in CIDMTR encoded a fourth putative MHC class I homolog, annotated *gp147.1*, that lies adjacent to three MHC class I homologs (*gp147*, *gp148*, and *gp149*) that were previously identified in 22122 (Figure 3c). 

The third region of discrepancy was located between nucleotides 230176 and 231697 of the 22122 sequence. Within this 1,522-bp region, 107 bps were missing from the CIDMTR genome, and the remainder correspond with a poorly conserved (51% average nucleotide conservation) 1,383-bp region in CIDMTR. This discrepant region partially overlaps *gp149* and results in CIDMTR- and 22129-encoded gp149 proteins that lack amino acid homology in residues 1-134 (22122) or 1-164 (CIDMTR) but are highly conserved within the remaining C-terminal 494 residues.

Finally, like 22122, the CIDMTR genome was found to have direct terminal repeat sequences, although the CIDMTR repeats were slightly shorter (841 nt) than those in 22122 (953 nt). 

**Figure 4:**
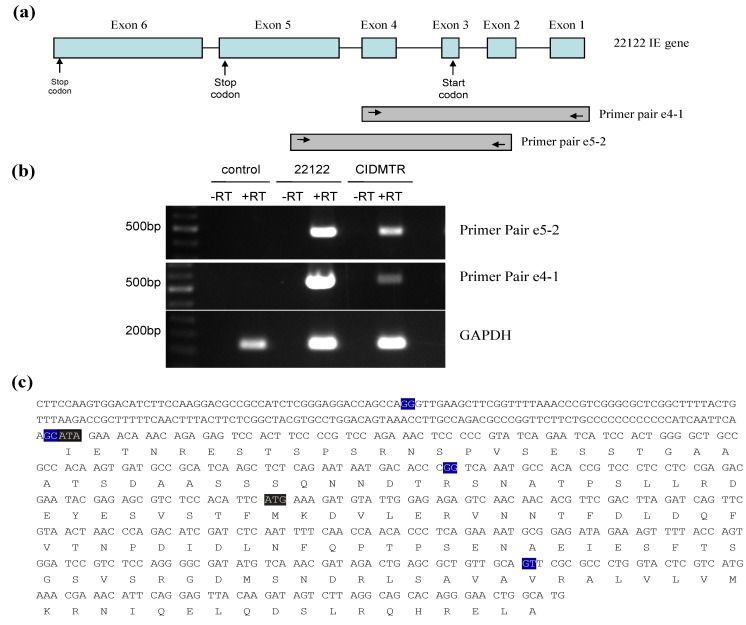
RT-PCR mapping of CIDMTR strain and 22122 strain splice sites. (**a**) Cartoon representation of the IE1/2 gene locus illustrating positions of primer pairs used for RT‑PCR. Introns are indicated by straight lines and exons are blue boxes, although exon 1 and 2 are non-coding. (**b**) Results of RT-PCR reactions e4-1, e5-2, and GAPDH (control) using RNA from uninfected cells or cells infected with 22122 or CIDMTR. (**c**) RT-PCR consensus sequence of CIDMTR strain. Exon junctions are highlighted in blue. The first gray highlighted sequence is an ATA codon; this is an ATG codon and the putative start codon for the 22122 IE1/2 proteins, but is not conserved in the CIDMTR sequence in tissue culture passaged virus. The second gray highlighted sequence may therefore represent the putative start codon for CIDMTR TC IE1/2.

Figure 3b highlights the regions of DNA sequence dissimilarity when the CIDMTR strain is compared to strain 22122. Figure 3c illustrates in map format the regions of genomic discontinuity. To confirm that these large-scale rearrangements did not arise during passage of virus in cell culture, PCR and sequence analyses were performed directly on both DNA purified from the original salivary gland homogenate (no tissue culture passage), and on DNA from the tissue culture-derived isolate (P_0_), with identical results (see Section 2.5 and Figure 4). The sequences of the PCR-generated fragments were identical to the Illumina/PacBio genome sequence, confirming that discontinuities with strain 22122 did not arise as an artifact of tissue culture passage. It therefore appears that, as it appears in its natural host, the CIDMTR strain lacks the *gp138.1* gene found in 22122. Similarly, 22122 lacks the fourth MHC class I homolog gene (*gp147.1*) found in CIDMTR. As the original SG extract from which 22122 was cultured is no longer available, it is not possible to conduct similar studies to determine whether *gp147.1* was absent from 22122 prior to its initial isolation in cell culture or was lost during subsequent *in vitro*/*in vivo* passage. Nevertheless, these results suggest that naturally occurring strains of GPCMV may be polymorphic with respect to the presence or absence of entire genes. Similar findings have been observed when comparing the Smith strain of murine cytomegalovirus to strains isolated from wild mice [37]. Further analyses of other primary virus isolates from “naturally infected” guinea pigs will be necessary to confirm this hypothesis and to determine if such polymorphisms are limited to specific genes, such as *gp138.1* and perhaps *gp147.1*, or include others.

**Figure 5:**
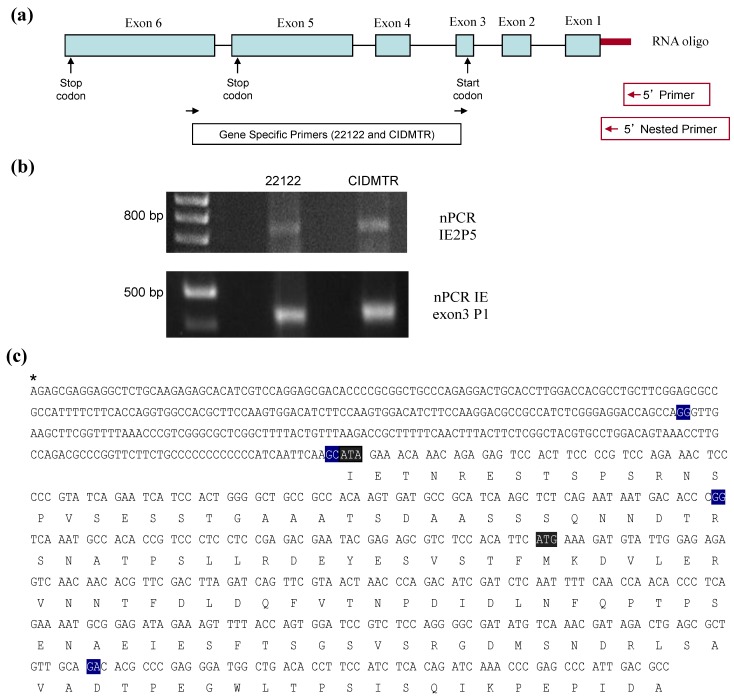
5' RACE analysis of CIDMTR IE region. (**a**) Schematic of IE gene indicating primers pair used in RACE. (**b**) nPCR gels for 22122 and CIDMTR using RNA purified under IE conditions. The CIDMTR RACE product is 734 nt compared to 729 nt for the 22122 strain, explaining its slightly higher migration by gel electrophoresis. (**c**) RACE consensus sequence of CIDMTR IE. Exon junctions highlighted in blue. First gray highlighted sequence is co-linear to the start codon for 22122; second highlighted sequence represents putative start codon for CIDMTR IE1/2.

Table 1 summarizes the predicted ORFs identified in the CIDMTR strain. ORFs that are highly conserved in cytomegaloviruses are noted in the table in upper case/bold font (e.g., **GP55**). In contrast, ORFs appearing unique to GPCMV are noted in lower case (e.g., gp138). The “C” designation in Table 1 refers to the complimentary strand. Splicing sites are predicted based on previously published reports and these need empiric confirmation in future studies unless otherwise indicated (Figure 4 and Figure 5); exons are described as coding exons. 

Amino acid sequences predicted from CIDMTR genes encoding conserved envelope glycoproteins were compared to those from strain 22122. There was striking sequence conservation of the gB homolog; although 13 SNPs were noted, only 2 coding changes were observed (overall identity of 99%). In contrast, sequence analyses of GP74 (gO) demonstrated the greatest degree of sequence divergence, with 303/374 (81% identity) noted, followed by GP75 (gH), with 613/726 (84%) identity compared to the 22122 strain. GP73, the homolog of HCMV glycoprotein N, a protein known to exhibit substantial sequence divergence across clinical isolates [19,38,39], demonstrated 92% identity between the CIDMTR and 22122 strains. These results confirm that CIDMTR represents a GPCMV strain distinct from 22122 as the amino acid sequences of gH, gO, and gN clearly diverged through gradual evolutionary processes and cannot be attributed to abrupt, limited events such as insertions, deletions, or duplications. This further implies that despite potential bottlenecks imposed by selective breeding, GPCMV, as it exists within commercial breeding colonies, exhibits significant strain diversity of a nature similar to that of HCMV. These findings suggest that GPCMV may provide a useful model to study the impact of naturally occurring strain variation on viral pathogenesis, particularly in the area of nonprimary infection and disease.

**Table 1:** GPCMV Strain CIDMTR Predicted ORFs.

GPCMV Open Reading Frames (ORFs): CIDMTR Strain					
ORF	Strand	Begin	End	Codons	Notes
gp1	C	12,464	12,769	101	GPCMV MIP 1α; CC chemokine homolog
gp2		14,840	15,685	281	Homology to MCMV M69^a^
gp3	C	17,197	19,563	788	Homology to THV T5^b^; US22 superfamily
gp4	C	20,829	21,152	107	Homology to RCMV r136^d^
gp5	C	26,721	27,818	365	Homology to MCMV m32^a^
**GP23**	C	33,299	34,501	400	UL23 homolog; US22 gene superfamily
**GP24**	C	34,739	35,956	405	UL24 homolog; US22 superfamily
**GP25**		36,542	38,194	550	UL25 homolog; tegument protein
**GP26**	C	38,360	39,043	227	UL26 homolog
**GP27**	C	39,166	41,211	681	UL27 homolog
**GP28**	C	41,311	42,378	355	UL28 homolog; US22 superfamily
**GP28.1**	C	42,866	44,287	473	UL28 homolog; US22 superfamily
**GP29**	C	44,653	46,623	656	UL29 homolog; US22 superfamily
gp29.1	C	47,247	47,861	204	US22 superfamily
**GP30**	C	49,082	50,800	572	UL30 homolog
**GP31**		51,094	52,572	492	UL31 homolog
**GP32**	C	52,665	54,365	566	UL32 homolog
**GP33**		54,585	55,868	427	UL33 homolog; 7-TMR GPCR superfamily
**GP34**		56,221	57,804	527	UL34 homolog
**GP35**		58,008	59,666	552	UL35 homolog
**GP37**	C	59,788	60,711	307	UL37 homolog
**GP38**	C	61,068	61,988	306	UL38 homolog
gp38.1	C	62,705	63,262	185	Positional homolog of HCMV UL40
gp38.2	C	63,620	64,933	437	Positional homolog of HCMV UL41a
gp38.3	C	65,624	66,478	284	Positional homolog of HCMV UL42
gp38.4	C	66,997	67,362	121	Homology to RCMV r42^d^
**GP43**	C	67,951	68,964	337	UL43 homolog
**GP44**	C	68,952	70,175	407	UL44 homolog
**GP45**	C	70,887	73,673	928	UL45 homolog
**GP46**	C	73,779	74,867	362	UL46 homolog
**GP47**		74,674	77,787	1037	UL47 homolog
**GP48**		77,784	84,155	2123	UL48 homolog
**GP48.2**	C	84,238	84,468	76	UL48a homolog
**GP49**	C	84,479	86,119	546	UL49 homolog
**GP50**	C	86,088	87,158	356	UL50 homolog
**GP51**	C	87,281	87,580	99	UL51 homolog; terminase subunit
**GP52**		87,900	89,480	526	UL52 homolog
**GP53**		89,473	90,459	328	UL53 homolog
gp53.1	C	90,196	90,537	113	Homology to RhCMV rh86; YP_068179.1
**GP54**	C	90,551	93,904	1117	UL54 homolog; DNA polymerase
**GP55**	C	93,947	96,652	901	UL55 homolog; glycoprotein B
**GP56**	C	96,549	98,816	755	UL56 homolog; terminase subunit
**GP57**	C	98,967	102,650	1227	UL57 homolog
gp57.1	C	104,411	104,926	171	Homolog to RCMV r23.1^d^
**GP69**	C	108,260	111,430	1056	UL69 homolog
**GP70**	C	112,136	115,339	1067	UL70 homolog; helicase-primase
**GP71**		115,338	116,114	258	UL71 homolog
**GP72**	C	116,277	117,350	357	UL72 homolog; dUTPase
**GP73**		117,432	117,833	133	UL73 homolog; glycoprotein N
GP73.5ex1		117,860	117,869	3	
GP73.5ex2		119,005	119,198	64	
**GP74**	C	117,780	118,904	374	UL74 homolog; glycoprotein O
**GP75**	C	119,349	121,529	726	UL75 homolog; glycoprotein H
**GP76**		121,694	122,533	279	UL76 homolog
**GP77**		122,247	124,091	614	UL77 homolog
**GP78**		124,473	125,717	414	UL78 homolog; 7-TMR GPCR superfamily
**GP79**	C	125,914	126,861	315	UL79 homolog
**GP80**		126,722	129,031	769	UL80 homolog; CMV protease
**GP80.5**		127,610	129,031	473	UL80.5
**GP82**	C	129,324	130,889	521	UL82 homolog; pp71
**GP83**	C	131,109	132,809	566	UL83 homolog; pp65
**GP84**	C	133,051	134,487	478	UL84 homolog
**GP85**	C	134,785	135,696	303	UL85 homolog
**GP86**	C	135,977	140,026	1349	UL86 homolog
**GP87**		140,407	143,328	973	UL87 homolog
**GP88**		143,231	144,499	422	UL88 homolog
**GP89ex2**	C	144,545	145,675	376	UL89 homolog; terminase subunit, exon 2
**GP91**		146,102	146,365	87	UL91 homolog
**GP92**		146,362	146,991	209	UL92 homolog
**GP93**		146,957	148,732	591	UL93 homolog
**GP94**		148,644	149,681	345	UL94 homolog
**GP89ex1**	C	150,032	150,913	291	UL89 homolog; terminase subunit, exon 1
**GP95**		150,967	152,235	422	UL95 homolog
**GP96**		152,468	152,830	120	UL96 homolog
**GP97**		152,910	154,727	605	UL97 homolog; protein kinase
**GP98**		154,747	156,531	594	UL98 homolog; alkaline nuclease
**GP99**		156,444	156,965	173	UL99 homolog; pp28
gp99.1		157,155	157,769	204	Homology to RCMV r4^d^
**GP100**	C	157,278	158,327	349	UL100 homolog; glycoprotein M
**GP102**		158,657	160,942	761	UL102 homolog
**GP103**	C	161,127	161,852	241	UL103 homolog
**GP104**	C	161,815	163,908	697	UL104 homolog; portal
**GP105**		163,748	166,531	927	UL105 homolog; helicase-primase
**GP112ex1**		176,745	177,498	315	UL112 homolog; replication accessory, ex 1
**GP112ex2**		177,606	177,782		UL112 homolog; replication accessory, ex 2
**GP112ex3**		178,115	178,131		UL122 homolog; replication accessory, ex 3
**GP114**	C	179,126	179,920	264	UL114 homolog; uracil glycosylase
**GP115**	C	179,986	180,762	258	UL115 homolog; glycoprotein L
**GP116**	C	180,755	181,654	299	Homology to THV t116^b^; Fc receptor//Ig
**GP117**	C	181,877	183,262	461	UL117 homolog
gp119.1	C	184,418	185,167	249	Similar to MCMV in ACE95619.1
**GP121.2**	C	185,160	185,834	224	Betaherpesvirus B7D8, accession AFK83957
**GP121.4**	C	186,299	187,174	291	UL121 homolog; Tupaia t121.4, NP_116476
**GP122ex3**	C	187,993	189,677	677	UL122 homolog; HCMV IE2
**GP122ex2**	C	191,079	191,311		
**^§^GP122ex1**	C	191,403	191,518		
**GP123ex3**	C	189,907	190,985	475	UL123 homolog; HCMV IE1
**GP123ex2**	C	191,079	191,311		
**^§^GP123ex1**	C	191,403	191,518		
**GP128**		195,400	196,455	351	Similar to Bat HSV B126; US22 Family
gp130		196,655	196,999	114	
GP129ex3	C	196,432	196,690	178	Homolog of HCMV UL128
GP129ex2		196,768	196,890		
GP129ex1	C	196,974	197,128		
GP131ex2	C	197,133	197,469	191	Homolog of HCMV UL130
GP131ex1	C	197,550	197,788		
GP133	C	197,788	198,174	128	Homolog of HCMV UL131
GP134	C	198,268	198,951	227	
gp138.2	C	199,367	200,875	502	
gp138.3	C	201,090	202,607	505	
gp139	C	202,706	204,793	695	THV T5; US22 superfamily
gp140		204,522	204,929	135	Homology to CCMV UL132
gp141	C	205,053	206,660	535	HCMV US23; US22 superfamily
gp142	C	206,928	208,622	564	HCMV US24; US22 superfamily
gp143	C	208,875	210,866	663	THV T5; US22 superfamily
gp144	C	211,109	213,403	764	US26; US22 gene superfamily
gp145	C	213,677	215,575	632	HCMV IRS1/TRS1; US22 superfamily
gp146	C	215,932	217,914	660	HCMV IRS1/TRS1; US22 superfamily
gp147.1	C	221,739	222,935	398	MHC class I homolog
gp147	C	223,124	224,218	364	MHC class I homolog
gp148	C	225,379	226,560	393	MHC class I homolog
gp149	C	228,236	230,209	657	MHC class I homolog
gp149.1		230,163	230,465	100	Unique ORF sequence in CIDMTR

**^§^** An observation of note is the codon, ATA, spanning nucleotides 191516–191518 (minus strand). This codon is ATG in strain 22122, which was annotated as the start codon for both IE2 (GP122; exons 3, 4 and 5) and IE1 (GP123; exons 3, 4 and 6) [42,43,44]. Whether alternative splicing is occurring in the CIDMTR strain, or the start codon for the IE1/IE2 proteins is different between the two strains of GPCMV (*i.e.*, in exon 4, not exon 3), requires further evaluation; this is described in greater detail in Section 2.4.

### 2.4. Sequence Characterization of DNA from Original SG Homogenate in Region of IE1/2 Start Codon

A surprising finding from the DNA sequence analysis was the finding of an ATA codon as the putative start codon of the IE1/IE2 protein product [42]. To examine this issue, sequence analyses of PCR-amplified DNA from the original salivary gland homogenates, as well as salivary gland homogenates from further *in vivo* passages, were undertaken. DNA from the original salivary gland homogenate that was the source of the CIDMTR isolate was compared to DNA from GPL cells infected with CIDMTR following cell culture passage. The PCR was done with the primer pair designated IE splice 3' P1 (5'-TGCGAAGCGATCTCTCTCAAC-3') and IE splice 5' P1 (5'-GTGGTTGTACGTGTCGTCGTCA-3'), which was predicted to produce an 864-bp product from CIDMTR DNA. The purified DNA was cloned and three clones from each reaction were sequenced. This analysis indicated that the original salivary gland source of the CIDMTR strain contained viral DNA that encoded an ATG codon (as does 22122), the putative start codon for the IE1/IE2 protein product, but that the tissue cultured-derived CIDMTR strain, following two passages in fibroblasts, had a DNA sequence corresponding to an ATA codon.

To further examine splicing of the IE region from the CIDMTR strain (tissue culture-derived; CIDMTR-TC) virus, reverse-transcriptase PCR was performed on RNA harvested at immediate early times post-infection. RNA was extracted from GPL cells infected with 22122 or CIDTMR at 4 hours post-inoculation cDNA was synthesized from 1 μg of total RNA. Conventional PCR was carried out using cDNA as template. PCR used primer pair IE exon4 F1 (5'-CCGCATTTTCTGAGGGTGTT-3') and IE exon1 R1 (5'-CATGCCAGTTCCCTGTGCTG-3'), and primer pair IE exon5 F1 (5'- GGAAGATGTCCACTTGGAAG-3') and IE exon2 R1 (5'- ACGTAGCCGAGAAGTAAAGT-3'). The expected product sizes for primer pair IE exon4 F1/ IE exon1 R1 and IE exon5 F1/IE exon2 R1 were 473 bp and 508 bp, respectively. 

A product of the expected size was purified, cloned into pCR2.1, and multiple clones with inserts from each reaction were sequenced. Notably, all CIDMTR-derived clones contained ATA at the putative (collinear) IE1/IE2 gene product start codon, confirming the DNA sequencing analysis. In contrast, all the 22122 clones contained an ATG at this position. The observed exon junctions were as described in the 22122 strain. The ATG codon in exon 3 was originally annotated as the putative IE1/2 start codon in 22122 [41] and the DNA sequence analysis of DNA from the CIDMTR strain purified directly from the salivary gland homogenate also demonstrated an ATG codon in exon 3. Thus, the finding of this ATA by DNA and RT-PCR sequencing in tissue culture-passaged CIDMTR virus was surprising. Since IE1/2 is abundantly expressed and ATA is known as an inefficient initiator of protein translation, we looked for ATGs that might serve as alternative start sites. Tissue culture‑passaged CIDMTR strain may employ a start codon in exon four for the IE1 and IE2 gene products (Figure 4), although the Kozak consensus sequence of this putative start codon (T at −3, A at +4) is relatively weak [45]. 

To further define IE transcription from the CIDMTR-TC virus, 5' RACE was performed. RNA was extracted and cDNA was synthesized with a specific sequence attached to the 5' UTR for capped mRNAs. After ligation of a specific RNA oligo to the 5'UTR of mRNAs, cDNA was synthesized using oligo-dT and random hexamers. The cDNA was used as template for a first round of PCR using GeneRacer 5' Primer and primer IE2 P5 (5'-GGCGTCAATGGGCTCGGGTTTGAT-3'). Two nested PCR reactions were then performed with the GeneRacer 5' Nested Primer, per the manufacturer’s specifications, and IE2 P5 or IE exon3 P1 (5'-GGCAGCCCCAGTGGATGATTCTGATA-3') (Figure 5b). Purified DNA was cloned and sequenced. Results matched the previously described splice sites for strain 22122 [42]. Using this primer combination, the RT-PCR product was 734 nt in size (5 nt longer than the homologous ATCC/22122 cDNA). The substitution of ATA for the putative ATCC/22122 IE2/3 start codon was once again noted. We did not observe other in-frame start codons for the CIDMTR strain IE1/2 other than the one identified in exon 4. Further experiments will be required to identify the start codon for IE1/2; possibly, both 22122 and CIDMTR strains use the start codon in exon 4, in spite of its suboptimal Kozak consensus. Alternatively, growth of the CIDMTR strain in fibroblasts may select for mutation of the exon 3 start codon, even upon minimal passage. Development of guinea pig epithelial/endothelial cell for isolation and passage of CIDMTR virus is an important future priority, to determine whether *in vivo* and *in vitro* passage result in different selective pressure on viral sequences.

### 2.5. PCR Confirmation of GPCMV-CIDMTR Genome Structure

**Figure 6:**
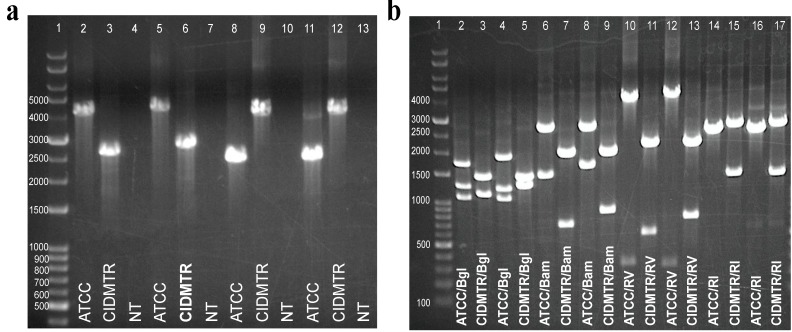
Strain-specific PCR assays differentiate ATCC 22122 strain of GPCMV from CIDMTR strain. (**a**) Primers were designed to amplify across discontinuous/unique regions as demonstrated in Figure 3b. Lane 1, kb ladder; lane 2, mismatch region 1 F1/R1, ATCC; lane 3, mismatch region 1, F1/R1, CIDMTR; lane 4, mismatch region 1, no template control; lane 5, mismatch region 1 F2/R2, ATCC; lane 6, mismatch region 1 F2/R2, CIDMTR; lane 7, mismatch region 1 F2/R2, no template control; lane 8, mismatch region 2 F1/R1, ATCC; lane 9, mismatch region 2 F1/R2, CIDMTR; lane 10, mismatch region 2 F1/R1, no template control; lane 11, mismatch region 2 F2/R2, ATCC; lane 12, mismatch region 2 F2/R2, CIDMTR; lane 13, mismatch region 2 F2/R2, no template control. (**b**) Restriction polymorphisms were as predicted by DNA sequence. PCR amplification products were digested with enzymes as indicated. Lane 1, kb ladder; lane 2, mismatch region 1 F1/R1 ATCC, *Bgl* 2; lane 3, mismatch region 1 F1/R1 CIDMTR, *Bgl 2*; lane 4, mismatch region 1 F2/R2 ATCC, *Bgl* 2; lane 5, mismatch region 1 F2/R2 CIDMTR, *Bgl* 2; lane 6, mismatch region 1 F1/R1 ATCC, *BamH* I; lane 7, mismatch region 1 F1/R1 CIDMTR, *BamH* I; lane 8, mismatch region 1 F2/R2 ATCC, *BamH* I; lane 9, mismatch region 1 F2/R2, *BamH* I; lane 10, mismatch region 1 F1R1 ATCC, *EcoR* V; lane 11, mismatch region 1 F1/R1 CIDMTR, *EcoR* V; lane 12, mismatch region 1 F2/R2 ATCC, *EcoR* V; lane 13, mismatch region 1 F2/R2, *EcoR* V; lane 14, mismatch region 2 F1R1 ATCC, *EcoR* I; lane 15, mismatch region 2 F1R1 CIDMTR, *EcoR* I; lane 16, mismatch region 2 F2/R2 ATCC, *EcoR* I; lane 17, mismatch region 2 F2/R2, *EcoR* I.

To confirm the structure of the CIDMTR strain compared to the 22122 strain, PCR was performed on viral DNA from both strains, using primers spanning the mismatched regions observed in the sequence analysis comparisons (Figure 3b, boxed/shaded regions). Two primers pairs were used for each region of mismatch (mismatch region 1 and mismatch region 2). PCR was performed using primer pairs mismatch-region 1 F1/R1 and mismatch-region 1 F2/R2, that amplify a ~4-kb region for the 22122 strain, but a ~2.5-kb region for the CIDMTR strain. The amplification region using primer pairs mismatch‑region 2 F1/R1 or mismatch-region 2 F2/R2 was predicted to be ~2.2 kb for the 22122 strain and ~3.7 kb for the CIDMTR strain. Primer sequences are indicated in Table 2. The results of these experiments confirmed that the genome configuration was precisely as predicted from the deep sequence analysis. Moreover, the PCR was also performed on DNA purified directly from the original salivary gland homogenate from which the CIDMTR isolate was obtained (data not shown). These results confirmed that the insertions and deletions identified by sequencing (Figure 3c) did not arise as an artifact of limited passage of virus in cell culture. The PCR results are shown in Figure 6. 

**Table 2:** Primer Sequences.

Primer Name	Primer Sequence
Mismatch Region 1 F1	5'-GTGAGACGTAAGAATAGCTTGC-3'
Mismatch Region 1 F2	5'-GATCCTTAGACTCTATCACGG-3'
Mismatch Region 1 R1	5'-GTGTTGTCACAATTGGCACATG-3'
Mismatch Region 1 R2	5'-ACATGGTCACGACAGAATC-3'
Mismatch Region 2 F1	5'-GTGGACAGGATCCCCAAATT-3'
Mismatch Region 2 F2	5'-CCAAATTTCTGTCGTCGGCG-3'
Mismatch Region 2 R1	5'-TGTTTCCGTGTCTGTCTCCGT-3'
Mismatch Region 2 R2	5'-GTCTTAGCCCGAGACCTTC-3'

### 2.6. Infection *In Vivo* and Development of a Real-Time PCR Assay for Detection of CIDMTR DNA

To evaluate DNAemia and end-organ infection in the course of *in vivo* studies, a strain-specific real-time PCR assay was developed in order to differentiate the GPCMV-CIDMTR strain from the 22122 strain. This assay focused on the amplification of sequences corresponding to the CIDMTR strain GP147.1 ORF (Table 1), since this sequence is absent in the 22122 strain. A GPCMV 147.1 specific PCR primer pair, consisting of CIDMTR147.1_464F (5'-ATGCAACATAGCGTGCTGAC-3') and CIDMTR147.1_583R (5'-GGGACAAAAGCACGATGAAC-3') was designed and utilized for the real-time PCR assay (described in detail in 3.5). These primers amplified a 120 bp region of the 147.1 gene specific for the CIDMTR strain. DNA was extracted from either 100 μL citrated blood or from fresh frozen tissues samples, as described in the Methods section. For quantitative PCR, both previously validated primers for the GP83 gene [sequences shared by both strains] and novel primers for the GPCMV *147.1* gene [sequences only found in the CIDMTR strain] were used for real‑time PCR assay. There was strong concordance between the viral load estimates identified by the gp147.1 and GP83 real-time primers (data not shown). As a negative control, GPCMV 147.1 primers were used in several PCR assays of 22122 DNA, with consistently negative results (data not shown).

Next, twelve young, GPCMV-seronegative outbred Hartley guinea pigs were divided into two groups of six/group. Each group was inoculated with CIDMTR strain virus (p1) at a dose of 1 × 10^5^ pfu, administered subcutaneously, as described in Section 3. Group 1 (n = 6) was treated with 200 mg/kg cyclophosphamide on day −1 and 50 mg/kg on day +6 following viral inoculation as described previously [32]; group 2 (n = 6) was sham-treated (PBS only). Whole blood and sera samples were collected on day 0, 3, 7, and 21. Animals were sacrificed on day 21 and tissue, including lung, liver, spleen, and brain, were collected for PCR analysis. In group 1, 5/6 animals were DNAemic, peaking at day 7 (mean, 3.2 +/− 0.3 log_10_ genomes/mL) while in group 2, the prevalence of DNAemia was lower (3/6 animals; 2.5 +/− 0.35 log_10_ genomes/mL, *p* = 0.06 compared to group 1). Viral DNA was most readily recovered from spleen upon dissection at day 21 post-infection. All 6 animals from each group had recoverable CIDMTR strain DNA in the spleen. Total spleen viral load in group 1 was 2.4 +/− 0.07 log_10_ copies/mg and was 2.4 +/− 0.1 log_10_ copies/mg in group 2 (p = NS compared to group 1). 

The ability of the CIDMTR strain to infect pregnant animals previously inoculated with the 22122 strain was next assessed. A total of 6 female seronegative Hartley guinea pigs received a primary infection with SG homogenate GPCMV (22122) at a dose of 1 × 10^4^ pfu, administered subcutaneously. A parallel group of animals were sham-treated (PBS only). Then, 5 weeks after inoculation, the animals in both groups were mated with GPCMV-seronegative breeders. Dams originally inoculated with the SG virus (22122 strain) were documented to undergo seroconversion; in addition, all were demonstrated to have low-level DNAemia after inoculation with 22122 virus (data not shown). Approximately 5 weeks after the initiation of mating, group 1 was challenged subcutaneously with CIDMTR strain at a titer of 6 × 10^5^ pfu. A total of 19 pups were born in the experimental group; all pups were liveborn. When pup tissues were analyzed, 4/19 (21%) of these animals had recoverable CIDMTR strain-specific DNA isolated from the salivary gland. One of these animals also had detectable DNA in the brain.

## 3. Experimental Section

### 3.1. Cells, Virus and DNA Preparation

GPCMV (strain 22122, ATCC VR682), and CPCMV/CIDMTR were propagated on guinea pig lung fibroblast cells (GPL; ATCC CCL 158) in F-12 medium supplemented with 10% fetal calf serum (FCS; Gibco-BRl), 10,000 IU of penicillin/liter, 10 mg of streptomycin/liter (Gibco-BRL), and 7.5% NaHCO3 (Gibco-BRL). When the P_1_ passaged CIDMTR virus exhibited extensive CPE in GPL cell culture, cells and virions were pelleted. Aliquots were fixed in phosphotungstic acid for EM studies (see below) and the remainder washed in 10 mM Tris (pH 8.0)/1 mM EDTA (TE), and lysis buffer (200 mM NaCl, 2% SDS, 200 μg/mL proteinase K, in TE), was added. Cells and virions were gently suspended, inverted, and incubated at 68 °C overnight. Following transfer to a 37 °C water bath, three successive phenol-chloroform extractions were performed, followed by ethanol precipitation.

### 3.2. Transmission Electron Microscopy

Cells were fixed in 1 mL of 2.5% glutaraldehyde in 0.1 M sodium cacodylate buffer and post fixed with 1% osmium tetroxide in 0.1 M sodium cacodylate buffer (all reagents from Electron Microscopy Sciences, Hatfield, PA, USA). After three washes in distilled water, samples were dehydrated using a 25%–100% ethyl alcohol gradient. Samples were then infiltrated with 2:1 ethanol: Embed 812 resin (Electron Microscopy Sciences, Hatfield, PA, USA) for 1 hour and subsequently transferred to a 1:2 ethanol: Embed 812 resin mixture for 1 hour. Cells were further infiltrated with 100% resin and were embedded and incubated at 58 °C for 24 hours to polymerize the resin. Embedded samples were trimmed and sectioned on a Leica UC6 Ultramicrotome (Leica Microsystems, Vienna, Austria). Thin sections (60–70 nm) were obtained and collected on a 200 mesh copper grid (Electron Microscopy Sciences, Hatfield, PA, USA) using a perfect loop. Grids were contrasted with 5% uranyl acetate for 20 minutes and Santos’ lead citrate for 6 minutes. For negative contrast, virions and dense bodies were collected from P_1_ supernatants of infected fibroblasts, transferred to airfuge tubes (Beckman-Coulter, Brea, CA, USA), and centrifuged at 30 PSI using an airfuge (Beckman-Coulter, Brea, CA, USA) for 20 minutes on parafilm and formvar coated copper grids (Electron Microscopy Sciences, Hatfield, PA, USA). Excess liquid was wicked and the grids were stained with 1% phosphotungstic acid for one minute. All sections were observed under JEOL 1200 EX II transmission electron microscope (JEOL LTD, Tokyo, Japan). Images were obtained using a Veleta 2K × 2K camera with iTEM software (Olympus SIS, Munster, Germany) [46]. 

### 3.3. Deep Sequencing and Sequence Analyses

For sequence analysis of CIDMTR viral DNA, virions were purified as described previously [44], and lysis buffer (200 mM NaCl, 2% SDS, and 200 µg/mL proteinase K in Tris-EDTA [TE]) was added. Following incubation at 68 °C overnight, three phenol-chloroform extractions were performed, followed by ethanol precipitation of viral DNA. Genomic sequencing was performed using Illumina MiSeq and Pacific Biosciences PacBio RS platforms. Approximately 5.2 million 151-bp paired-end MiSeq reads were generated at the University of Minnesota’s Biomedical Genomics Center with a nominal insert size of 400 bp. Removal of low quality reads and PhiX sequence resulted in a set of 4.0 million cleaned reads, approximately 11,000× coverage. Initial scaffolds were generated from the cleaned Illumina reads using the ABySS assembler (version 1.3.4) [47]. Scaffold quality was assessed manually by comparison with the reference strain, 22122 [44,48,49], and by remapping the reads using Bowtie 2 and scrutinizing local coverage and consensus using Tablet [50] and SAMtools [51]. Special attention was paid to correct alignment and orientation of the paired ends. Regions of weak coverage and scaffold gaps were identified and closed either by manual local assembly [52] or by Sanger sequencing. Independent validation of the pseudomolecule was also performed using the longer PacBio RS reads, validating the Sanger sequencing and manual assembly. SMRT Analysis software [53] produced 998 high quality (“corrected”) reads ranging between 509–15,898 bp, median 6,257 bp (approximately 27× coverage), as well as another set of scaffolds. These data were used to evaluate the Illumina assembly, specifically its structural correctness, and to correct misassembled repeat regions. The resulting complete genome was deposited with the EMBL Nucleotide Sequence Database (accession number HG531783).

### 3.4. RT-PCR and RACE Analyses

Reverse-transcriptase PCR was performed on RNA harvested at immediate early times post‑infection. RNA was extracted from GPL cells infected with 22122 or CIDTMR, at 4 hours post‑inoculation, using the RNeasy mini kit (Qiagen, Hilden, North Rhine-Westphalia, Germany) according to the manufacturer’s instructions. RNA was treated with RNase-free DNase Set (Qiagen) while in the column according to manufacturer’s instructions. cDNA was synthesized from 1 μg of total RNA using Quantitect Reverse Transcription kit (Qiagen). Conventional PCR was carried out using cDNA as template and AmpliTaq Gold Fast PCR Master Mix (Invitrogen, Carlsbad, CA, USA). Primers are as described in Section 2.4. The PCR products were run in a 0.7% agarose gel. A band of the expected size was cut form the gel and purified using QIAquick Gel Extraction Kit (Qiagen). The purified DNA was cloned into pCR2.1 (Invitrogen) using the TA Cloning Kit (Invitrogen). Three of clones with inserts from each reaction were sequenced with T3 promoter, M13 Reverse and the PCR primers. 

For 5' RACE analysis, RNA was used as template for cDNA synthesis using the GeneRacer kit (Invitrogen) in order to generate a cDNA with a specific sequence attached to the 5' UTR for only mRNAs that were capped. After ligation of a specific RNA oligo to the 5'UTR of mRNAs, cDNA was synthesized using a combination of oligo-dT and random hexamers. The cDNA was used as template for a first round of PCR using GeneRacer 5' Primer and primer IE2 P5 (5'-GGCGTCAATGGGCTCGGGTTTGAT-3'). In order to increase the specificity and quantity of the PCR product, nested PCR (nPCR) was performed in a dilution (1:20) of the PCR product. Two nPCR reactions were performed with the GeneRacer 5' Nested Primer, per the manufacturer’s specifications, and IE2 P5 or IE exon3 P1 (5'- GGCAGCCCCAGTGGATGATTCTGATA-3'). A band of the expected size was cut form the gel and purified using QIAquick Gel Extraction Kit (Qiagen). The purified DNA was cloned into pCR4-TOPO (Invitrogen) using the TOPO TA Cloning. Clones were analyzed for the present of the insert by digestion with *EcoR* I. Three of the clones with insert from each reaction were sequenced with T3 promoter, M13 forward (−20) and the PCR primers. 

### 3.5. GPCMV-CIDMTR Strain PCR Assay

For confirmation of viral genome structural differences noted by deep sequencing, PCR was performed for each virus using primers indicated in Table 2. The PCR reaction was performed in a 50 μL of total volume using GoTaq long PCR Master Mix from Promega and 1.0 μM primers. The DNA template was total genomic and viral DNA extracted from GPL cells infected with 22122 or CIDMTR tissue culture strain. The conditions for the PCR were: initial denaturation at 95 °C for 2 min, followed by 95 °C for 30 s, 53 °C for 30 s, 72 °C for 4 min for a total of 35 cycles, and elongation at 72 °C for 10 min. The PCR product (4 μL) was subjected to electrophoresis in a 0.7% agarose gel. The PCR product from mismatch 1 F2/R2 and mismatch 2 F2/R2 were purified from the gel using the Geneclean**^®^** II kit (MP Biomedicals, Santa Ana, CA, USA) following the manufacturer’s instructions. The cleaned PCR product was then sequenced by Sanger sequencing (Functional Biosciences, Madison, WI, USA). Purified PCR products were also subjected to restriction endonuclease comparisons as indicated in Figure 5b.

The real-time PCR assay focused on the amplification of sequences corresponding to the CIDMTR strain gp147.1 ORF (Table 1), since this sequence is absent in the 22122 strain sequence. A GPCMV gp147.1 specific real-time PCR primer pair, consisting of CIDMTR147.1_464F (5'-ATGCAACATAGCGTGCTGAC-3') and CIDMTR147.1_583R (5'-GGGACAAAAGCACGATGAAC-3') was designed and utilized for the real-time PCR assay. These primers amplified a 120 bp region of the *gp147.1* gene specific for the CIDMTR strain. The specific hydrolysis probe used for detection was CIDMTR147.1_494P (FAM-GTGTTCGTGTCCTTGATCGTACGCA-BHQ1). A second GPCMV 147.1 specific primer pair, CIDMTR147.1_225F (5'-AATGGTTCGCTACGGACATC-3') and CIDMTR147.1_368R (5'-CGGACAACGGAACATACTTG-3') was also utilized in real-time PCR assays. These primers amplified a 144 bp region of 147.1 specific for the CIDMTR strain. The specific hydrolysis probe used for detection with this primer pair was CIDMTR147.1_262P (FAM‑TTCCTCGACGAAGCTCGCGGTATAAT-BHQ1). In each instance, the PCR reactions were performed in a 25 μL reaction, using LightCycler 480 Probes Master from Roche (Penzberg, Bavaria, Germany); as well as 0.4 μM primers, 0.1 μM probe and 0.4 u/μL of UNG. PCR was performed using the LightCycler 480 Real-Time PCR System (Roche) under the following conditions: initial denaturation at 95 °C for 10 min, followed by 95 °C for 10 s, 56 °C for 15 s, 72 °C for 10 s for a total of 45 cycles, then a final hold step at 40 °C. The first primer pair (464F and 583R) was chosen for detection of viral genome for *in vivo* studies. Data were analyzed with the LightCycler Data Analysis Software (version 1.5; Roche) [54] using standard curves generated using serial dilutions of plasmid pCR2.1 with *gp147.1* at known concentrations. Negative results were arbitrarily assigned a level of 50 for the purpose of statistical comparisons, based upon limit-of-detection analyses observed in other real-time PCR experiments [55].

### 3.6. Animal Challenge Studies

All animal studies were performed with the approval of the University of Minnesota Institutional Animal Care and Use Committee (IACUC). Some animals were immune suppressed at day −1 (200 mg/kg) and day +6 (50 mg/kg) with cyclophosphamide delivered by intraperitoneal injection. For experiments described in Section 2.6, both cyclophosphamide-treated and untreated animals (n = 6/group) were challenged with CIDMTR strain virus (P_1_ workpool) at a dose of 1 × 10^5^ pfu by subcutaneous injection. Blood samples were collected at day 0, 3, 7 and 21 post-inoculation and animals humanely sacrificed at day 21 for collection of tissue samples for PCR analyses. Pregnancy/challenge studies were conducted as described in Section 2.6. Liveborn pups were sacrificed within 72 hours of delivery for DNA extraction and subsequent PCR.

## 4. Conclusions

The 22122 strain was originally isolated by Hartley in 1957 [27]. As the only characterized isolate of GPCMV it was used in virtually all subsequent GPCMV research. However, GPCMV infection is common among animals in commercial breeding colonies. In a longitudinal study conducted by Hsiung and colleagues from 1974 to 1979, GPCMV-neutralizing antibody was observed in 25% of Hartley strain guinea pigs obtained from commercial sources while virus was isolated from only 6 of 204 animals [56]. The percentages of antibody-positive animals obtained from different sources varied from shipment to shipment, ranging from 8%–50%. It does not appear that any of these viral isolates were retained. Thus, given only a single characterized isolate, the extent to which diverse GPCMV strains have been maintained within these domesticated populations was not previously known. Given that Hartley guinea pigs are descended from animals imported from South America to Europe in the 16th century, and that these animals subsequently underwent centuries of selective breeding, first as pets and later as research animals, the possibility existed that bottlenecks in the breeding history of Hartley guinea pigs could have limited the genetic diversity of GPCMV strains currently endemic in commercial breeding facilities. 

In the current study, a second virus was independently isolated and characterized, again from a Hartley strain guinea pig obtained from a commercial supplier in the United States. Based on a high degree of genetic relatedness to 22122, predominantly similar restriction endonuclease patterns, and an conserved overall genomic structure, we conclude that CIDMTR is a GPCMV and not a novel and distinct betaherpesvirus. However, based on restriction pattern polymorphisms and significant divergence of amino acid sequences for several envelope glycoproteins (particularly gH and gO), we conclude that CIDMTR and 22122 represent two distinct strains of GPCMV and are not minor variants of the same strain. This further suggests that GPCMV strain diversity has been sustained within commercial breeding colonies and that other strains exhibiting similar levels of divergence may exist and could potentially be exploited to further extend this important animal model. 

The passage history of 22122 is uncertain. In the original report by Hartley, two parallel isolations of GPCMV were reported: one, from salivary gland homogenate obtained from guinea pigs purchased by a commercial supplier in Yonkers, NY, and a second isolate obtained from a second supplier in New York state. Reactivation of virus may have been driven by allogeneic responses engendered from injecting SG extract into an animal with a different allotype, as has been suggested may drive HCMV and MCMV reactivation during transplantation [57,58]. Allogeneic responses may similarly have played a role, along with cyclophosphamide immunosuppression, in reactivation of the CIDMTR strain, since repeated tissue culture of salivary gland explants directly obtained from seropositive animals failed to result in growth of virus (data not shown). With respect to the original isolation of 22122, it is worth noting that serial *in vivo* passages may have occurred in the context, for some animals, of “mixed infection”, since Hartley noted that 7.7% (3/39) of “control NIH” strain guinea pigs aged 5 months or older were naturally infected, as evidenced by infrequent salivary gland inclusions [27]. This strain underwent 22 additional passages in cell culture of fibroblasts, and appears to be the strain that eventually was deposited with ATCC. The precise date of deposit is unclear, but reports of GPCMV research from the 1960s [59] and early 1970s describe obtaining this strain directly from the NIH, while studies since the late 1970s describe obtaining the virus from ATCC [60]. Between its original isolation by Hartley and its submission to ATCC, it appears to have undergone 54 additional passages in guinea pigs, and six additional passages in cell culture (3 passages in guinea pig embryo fibroblasts, and 3 passages in CCL 158 cells [61]. The 22122 strain derives from multiple rounds of both *in vivo* passage, some possibly occurring in the context of mixed infection, and 25–30 passages in cell culture. Thus, it is possible that 22122 underwent changes after isolation from the initial animal, either in cell culture or during *in vivo* passage. 

In contrast, CIDMTR was subjected to minimal passage (one passage *in vivo* and two passages in cell culture) prior to genomic characterization. Thus, the genomic structure and sequence of the CIDMTR strain may more likely represent a *bona fide* “wild-type” GPCMV sequence than does the 22122 strain. 

Sequence comparison with the 22122 strain revealed generally good conservation of protein coding sequences, although three areas of substantial discontinuity were noted. Thus, each strain contains unique sequences that can be used as markers to distinguish the strains during *in vivo* coinfection experiments. One of the regions unique to CIDMTR contains an ORF encoding a fourth putative MHC class I homolog not found in the 22122 strain. Since the three putative MHC class I homologs found in 22122 appear to be important for the *in vivo* pathogenesis of infection [55], further functional comparisons of the two strains will be of interest. In spite of minimal cell culture passage, at least one mutation, in the IE1/IE2 start codon, was observed in the tissue culture-adapted CIDMTR virus, compared to sequences amplified from salivary gland homogenate. This observation is similar to reported nucleotide sequence comparisons between several open reading frames in the DNA of different laboratory-adapted strains and clinical isolates of HCMV that has revealed amino-terminal sequence extensions of ORFs with alternate start codon usage [62]. Sequence differences with respect to start codon usage have also been noted upon comparison of laboratory-passaged and “wild” isolates of MCMV [37]. Future sequence analysis of CIDMTR DNA propagated solely *in vivo* is therefore warranted. Studies in immune competent and immune compromised guinea pigs confirm the ability of the CIDMTR virus to disseminate and produce viremia (DNAemia). Variation in glycoprotein protein coding sequences were noted, particularly for the gH and gO proteins, suggesting that this virus may be useful for the study of re-infection of immune guinea pigs in the guinea pig model of congenital cytomegalovirus infection. Cross-neutralization studies examining strain-specific antibody responses to envelope glycoproteins would enhance the usefulness of this new strain for the modeling of vaccine-mediated protection against re-infection and congenital transmission in this uniquely valuable model.

## EMBL Accession Number

The genome sequence for this strain has been deposited with the EMBL Nucleotide Sequence Database under the accession number HG531783. The CIDMTR strain has been deposited with ATCC (Rockville, MD, USA; accession number PTA-120714), or is available upon request from the corresponding author.
